# Good response to PAH-targeted drugs in a PVOD patient carrying Biallelic EIF2AK4 mutation

**DOI:** 10.1186/s12931-018-0900-2

**Published:** 2018-10-01

**Authors:** Li Liang, Hua Su, Xiuqing Ma, Ruifeng Zhang

**Affiliations:** Department of Respiratory medicine, Sir Run Run Shaw Hospital, Medical School of Zhejiang University, Hangzhou, China

## Abstract

Pulmonary veno-occlusive disease (PVOD) is a rare and fatal cause of pulmonary arterial hypertension (PAH). Different from other types of PAH, PVOD patients have a dismal prognosis because of the progressive nature of pulmonary vascular involvement and fatal pulmonary edema induced by PAH-targeted drugs. Lung transplantation is the only choice for these patients. In a recent article published in the journal, Yang and his colleagues found pulmonary edema was not demonstrated in 2 of the 6 PVOD patients injected with prostacyclin analogues (a kind of PAH-targeted drug). Regretfully, none of these 6 patients underwent microscopic examination of lung tissues. Here, we reported a sporadic PVOD patient evidenced by pathology and EIF2AK4 biallelic mutation. The patient was followed over the course of 3 years in our center. During the 3 years, he was admitted into our hospital for many times for the acute exacerbation of pulmonary hypertension. However, after treatment with many kinds of PAH-targeted drugs, the pulmonary hypertension was in control and he feel better every time. The present patient displayed different treatment response comparing with previous reports. It suggests that PVOD is a heterogeneity population and different patients have different characteristics including clinical manifestation, genomics, treatment response et al. How to pick off this portion of patients timely is the core issue. Lots of important works are necessary to answer this question. However, we can see a glimmer of hope form this patient at least.

**Electronic supplementary material:**

The online version of this article (10.1186/s12931-018-0900-2) contains supplementary material, which is available to authorized users.

To the editor:

Yang, and his colleagues, did a genetic analysis in a cohort of 191 PAH (pulmonary arterial hypertension) patients [1]. In their study, six patients were initially diagnosed with IPAH (idiopathic pulmonary arterial hypertension), but after homozygous or compound heterozygous EIF2AK4 (eukaryotic translation initiation factor 2 a kinase 4) mutations were detected in these patients, their diagnosis was corrected to PVOD (pulmonary veno-occlusive disease). Pulmonary edema was not demonstrated in 2 PVOD patients injected with prostacyclin analogues. None of these 6 patients underwent microscopic examination of lung tissues [1]. In another study, PVOD patients showed no response to PAH target therapy and had the risk of acute pulmonary edema induced by PAH-targeted drugs [2]. However, the authors showed different results [1]. In our study, we detailed the management of one patient with sporadic PVOD over the course of 3 years. PVOD was diagnosed by pathology and EIF2AK4 biallelic mutation. In the 3 years that we followed the patient he was admitted into our hospital multiple times for the acute exacerbation of pulmonary hypertension. The pulmonary hypertension was under control and the patient reported improvement after pulmonary vasodilator treatment.

We reported the case of a 51-year-old man complaining of dyspnea on exertion for 2 years and progressive dyspnea for 1 week in July 2015. He was a cook. The patient had no family history of pulmonary hypertension or other heart and lung diseases. In July 2015, he diagnosed as a functional class III (New York Heart Association (NYHA)) with the 6-min-walk distance of 475 m. Arterial blood gases test indicated that PaO_2_ was 55.9 mmHg and PaCO_2_ was 34.5 mmHg in breathing room air. The concentration of NT-proBNP (N-terminal pro-brain natriuretic peptide) was 3590 ng/mL. The results of hematologic, biochemical tests, thyroid function test, antinuclear antibody, antiphospholipid antibody, and anti-vasculitis antibody were within the normal range. The transthoracic echocardiogram revealed a dilated right ventricle and an estimated right ventricular systolic peak pressure of 142 mmHg. Pulmonary function test showed mild dysfuction of carbon monoxide diffusing capacity dysfunction (60% as predicted). Chest CT revealed ground-glass opacities, septal line thickening, and enlarged mediastinal lymph nodes (Fig. 1a-b). The ventilation/perfusion lung scan was normal. Right heart catheterization at rest showed severe pulmonary arterial hypertension with mean pulmonary artery pressure at 76 mmHg, pulmonary artery wedge pressure at 14 mmHg, pulmonary vascular resistance at 3.2 Wood units, and cardiac output 2.72 L/min. After multidisciplinary discussion, a CT-guided percutaneous lung biopsy was performed, showing occlusion of the pulmonary small vein (Fig. 1c). Genetic test demonstrated biallelic EIF2AK4 mutation in c.1392delT (p.Arg465fs) and other mutations (c.99 T > C (p.Ile33=); c.1524A > T (p.Leu508Phe); c.2750A > G (p.Lys916Arg); c.2757A > G (p.Ala919=)). Over the 3 year period, the patient was admitted into our hospital multiple times for the acute exacerbation of pulmonary hypertension. He has a good response to PAH-targeted drugs (Table 1). No signs of pulmonary edema were found. Lung CT scan was repeated and pulmonary edema was not detected. The detailed treatment course of the PVOD patient is seen in Additional file 1.

**Fig. 1:**
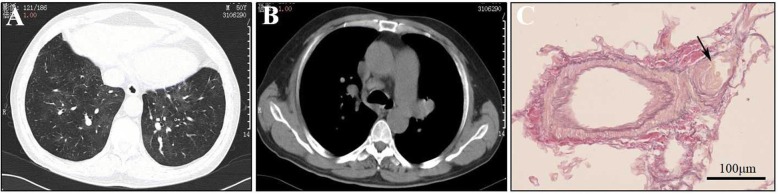
Representative chest CT and histopathological images obtained in a biallelic EIF2AK4 mutation PVOD patient. **a, b** Chest CT revealed ground-glass opacities, interlobular septal thickening, and enlarged mediastinal lymph nodes. **c** Hematoxylin–eosin staining of the lung specimen. Occlusion of the pulmonary small vein was indicated by the arrow

**Table 1:** Summarized of the treatment course of the PVOD patient

Time of Admission	2015.7	2016.3	2017.3	2018.1	2018.4
SaO_2_(pre-treatment, 21% O_2_)	88	89	90	88	89
SaO_2_(%) (post-treatment, 21% O_2_)	96	95	96	94	96
NT-proBNP (ng/ml, pre-treatment	3590	5080	24,389	9423	3957
NT-proBNP (ng/ml, post-treatment	980	746	890	1020	920
Functional class (NYHA, pre-treatment)	III	III	III	IV	III
Functional class (NYHA, post-treatment)	II	II	II	II	II
PAH-targeted drugs (in hospital)	Bosentan	Ambrisentan+Sildenafil	Ambrisentan+Sildenafil	Ambrisentan+Sildenafil+Treprostinil injection	Ambrisentan+Sildenafil+Treprostinil injection
PAH-targeted drugs (discharge)	Bosentan (changed to Ambrisentan, because of liver dysfunction 4 months later), Ambrisentan	Ambrisentan+Sildenafil	Ambrisentan+Sildenafil	Ambrisentan+Sildenafil	Ambrisentan+Sildenafil

Note: *SaO*_*2*_ oxygen saturation, *NT-proBNP* N-terminal pro-brain natriuretic peptide, *NYHA* New York Heart Association

PVOD is a rare and often fatal cause of pulmonary arterial hypertension. It is categorized into a separate PAH-related group in the current classification of pulmonary hypertension [3]. PVOD is histologically characterized by widespread fibrous intimal proliferation of septal veins and preseptal venules, and is frequently associated with pulmonary capillary dilatation and proliferation [4]. It shares several clinical and hemodynamic similarities with IPAH, thus often leading to a misdiagnosis as IPAH [2]. Unlike IPAH, PVOD patients have a dismal prognosis because of the degenerative nature of pulmonary vascular condition and the lack of response to pulmonary vasodilators. Lung transplantation is often the only option for these patients [5].

This specific case is unique because it included study of clinical diagnosis, radiological and histological examination, genetic test and vasodilators response over a 3 year period. The patient was a sporadic PVOD, and his family members had no history of pulmonary hypertension. Occupational exposure to organic solvents is a novel risk factor for PVOD [6]. Since he worked as a cook over 30 years, long-term exposure to organic solvents may have been a risk factor. Apart from common PVOD treatment, the patient had a good response to major target drugs. Nossent and his colleagues reported that in addition to typical veno-occlusive lesions, substantial pulmonary arterial lesions and important microvascular remodeling were also found in 24 cases of PVOD [7]. These PVOD patients had pathological characteristics similar to PAH. Hence, we posited these similarities may contribute to the good response to vasodilators in our patient. We observed typical veno-occlusive lesions in the lung biopsy specimen of our patient; however, other pulmonary arterial and microvascular lesions were not evident. We posited that this was because of the limited size of the percutaneous lung biopsy specimen which provided insufficient data. Thoracotomy or thoracoscopic surgery cannot be tolerated by this patient. So, percutaneous lung biopsy was performed by CT-guided. Biallelic EIF2AK4 mutation was also detected in this patient. The presence of a biallelic EIF2AK4 mutation is sufficient to confirm the diagnosis of PVOD [3]. Girerd and his colleagues reported that biallelic EIF2AK4 mutations were found in all familial cases of PVOD; however, mutations were identified only in 9% of sporadic PVOD patients [8]. We detected biallelic EIF2AK4 mutation in c.1392delT (p. Arg465fs) in our patient, which was consistent with the previous report in an inherited pulmonary capillary hemangiomatosis family [9]. However, other non-biallelic mutations were also detected. The significance of these non-biallelic mutations in PVOD is unclear. We posited that these mutations predict the good response to PAH-targeted drugs for PVOD.

## Conclusion

PVOD has no cure, and patients should undergo a lung transplant. Specific pulmonary vasodilators are contraindicant for these patients, because of poor response to the drugs and a following fatal pulmonary edema. Our patient displayed different treatment responses than previous studies, suggesting that PVOD patients are heterogeneous with differentent characteristics including clinical manifestation, genomics, treatment response. How to pick off this portion of patients timely is the core issue. Further study is necessary to answer this question. However, the current results are hopeful.

## Additional file

Additional file 1: The detailed treatment course of the PVOD patient is below. (DOC 25 kb)

## Abbreviations

EIF2AK4: Eukaryotic translation initiation factor 2 a kinase 4
IPAH: Idiopathic pulmonary arterial hypertension
NT-proBNP: N-terminal pro-brain natriuretic peptide
NYHA: New York Heart Association
PAH: Pulmonary arterial hypertension
PVOD: Pulmonary veno-occlusive disease
