# Supplementary material for: Good response to PAH-targeted drugs in a PVOD patient carrying Biallelic EIF2AK4 mutation
Source: Respir Res. 2018 Oct 1;19:192. doi: 10.1186/s12931-018-0900-2 (PMC6167821; doi:10.1186/s12931-018-0900-2)
Supplement: Supplementary file 1 — The detailed treatment course of the PVOD patient is below. (DOC 25 kb) [file 12931_2018_900_MOESM1_ESM.doc]

**Additional file 1: The detailed treatment course of the PVOD patient is below.**

In the first time admission into our hospital (July, 2015), we gave him Bosentan(62.5mg PO q12h, first 4 weeks; 125mg PO q12h, 4 weeks later) firstly, when we finished all examinations. The patient got better 2 weeks later, and we let him discharge. After discharge, Bosentan was continuously used. The liver function was monitored every week. However, 4 months later, liver dysfunction was detected (transaminase level was increased more than 10 folds comparing to normal range). We considered this maybe due to the liver injury of Bosentan. So, we transfered Bosentan to Ambrisentan(5mg PO qd). Transaminase level decreased to normal range after Bosentan was stopped. Ambrisentan was continuously used. The patient was stable until the second admission into hospital.

The patient was admitted into our hospital for the second time (March, 2016) because of the acute exacerbation of dyspnea. After full examination, we considered worsen of PVOD. On the base of Ambrisentan, Sildenafil(20mg PO q8h) was added. The patient got better about 10 days later and we let him discharge. Ambrisentan and Sildenafil were continuously used after discharge. The patient was stable until the third admission into hospital.

The patient was admitted into our hospital for the third time (March, 2017) because of the acute exacerbation of dyspnea. After full examination, we considered worsen of PVOD also. On the base of Ambrisentan and Sildenafil, we suggested Treprostinil should be added. However, the patient refused to use Treprostinil (because of the high cost of Treprostinil). We gave him diuretics and other supportive care, besides Ambrisentan and Sildenafil. Fortunately, he got better 3 weeks later and we let him discharge. Ambrisentan and Sildenafil were continuously used after discharge. The patient was stable until the fourth admission into hospital.

The patient was admitted into our hospital for the fourth time (January, 2018) because of the acute exacerbation of dyspnea. After full examination, we considered worsen of PVOD also. On the base of Ambrisentan and Sildenafil, we suggested Treprostinil should be added also. In this time, Treprostinil (injection, up to 20ng/kg/min) was used by the agreement from the patient. The patient got better about 2 weeks later and we let him discharge. We suggested that Treprostinil should be used continuously. However, the patient refused to use Treprostinil after discharge. Ambrisentan and Sildenafil were continuously used after discharge. The patient was stable until the fifth admission into hospital.

The patient was admitted into our hospital for the fifth time (April, 2018) because of the acute exacerbation of dyspnea. After full examination, we considered worsen of PVOD also. Besides Ambrisentan and Sildenafil, Treprostinil (injection, up to 20ng/kg/min) was added in hospital. The patient got better 2 weeks later and we let him discharge. We suggested that Treprostinil should be used continuously and the patient refused to use Treprostinil again after discharge. Ambrisentan and Sildenafil were continuously used after discharge. The patient is stable up to now.
